# Prognostic value of KIT/PDGFRA mutations in gastrointestinal stromal tumors: a meta-analysis

**DOI:** 10.1186/1477-7819-12-71

**Published:** 2014-03-28

**Authors:** Liang Zong, Ping Chen

**Affiliations:** 1 Department of Gastrointestinal Surgery, Su Bei People’s Hospital of Jiangsu Province, Yangzhou University, Yangzhou, Jiangsu Province 225001, China; 2 Department of Gastrointestinal Surgery, Graduate School of Medicine, University of Tokyo, 7-3-1 Hongo, Bunkyo-ku, Tokyo 113-8655, Japan

**Keywords:** Gastrointestinal stromal tumors, KIT, PDGFRA, Prognosis, Meta-analysis

## Abstract

**Background:**

The postulated relationship between KIT/PDGFRA mutations and their prognostic value in gastrointestinal stromal tumors (GISTs) has generated intense attention during the past decade, despite the fact that a great deal of studies have been conducted on this subject. To provide a strong quantitative estimate of this postulated relationship, we carried out a meta-analysis which combined, compared, and summarized the results of existing relevant studies.

**Methods:**

Studies were identified by searching databases and reviewing citations in relevant articles. Of 48 potentially relevant studies, we combined individual patient data from 18 studies which involved 1,487 patients with GISTs, by which we made a comparison between the positive KIT mutation subgroup and the negative KIT mutation subgroup (PDGFRA mutation and wild type). We tabulated and analyzed the patient characteristics from each study, including general information such as age and gender, histopathological parameters, and clinical follow-up outcomes.

**Results:**

KIT mutations, compared with PDGFRA mutations and wild type, showed a marked increased risk not only for tumor size (>5 cm) but also for higher mitotic activity (>5), suggesting that KIT mutations significantly correlated with the National Comprehensive Cancer Network (NCCN) high risk or National Institutes of Health (NIH) high risk (1.74 (95% CI, 1.20 to 2.53) and 2.00 (95% CI, 1.08 to 3.68), respectively). Moreover, higher recurrence and metastasis was observed in GISTs with KIT mutations, revealing its closer correlation with clinical malignant risk (*P* <0.001 for each, with odds ratio (OR) of 2.06 (95%, 1.37 to 3.11) and 2.77 (95%, 1.64 to 4.67), respectively). High risk or malignant GISTs with KIT mutations had a significantly poorer prognosis, as measured by 3-year overall survival, compared to those with PDGFRA mutations and wild type (0.47 (95% CI, 0.25 to 0.90)).

**Conclusions:**

KIT mutations, compared with PDGFRA mutations and wild type, represent a poorer prognostic marker in high risk or malignant GISTs.

## Background

Gastrointestinal stromal tumors (GISTs) are rare tumors, but are the most common primary mesenchymal tumor of the gastrointestinal tract [1]. GISTs express the tyrosine kinase receptor, KIT, which is the protein product of the KIT protooncogene. GISTs are generally characterized by gain-of-function mutations of KIT [2], and less often by PDGFRA or BRAF gene mutations [3-5]. In fact, the frequency of KIT/PDGFRA mutations in GISTs varies due to sample size, race, and geographic area. Around 85% of GISTs harbor mutations in KIT or PDGFRA [6].

To our knowledge, GISTs have a wide spectrum of biological behaviors ranging from benign to malignant. Due to the tumor’s specific biological behavior, there is no standard definition of benignity and malignancy when a patient is diagnosed with GIST at an early stage. In 2001, the National Institutes of Health (NIH) recommended the use of risk assessment in predicting GIST behavior, in preference to trying to distinguish between benign and malignant lesions. They categorized GISTs into four groups on the basis of the combined parameters of tumor size and mitotic count, as follows: very low risk, low risk, intermediate risk, and high risk [7]. In 2006, another risk system for malignancy adding tumor site was established by the National Comprehensive Cancer Network (NCCN) criteria, based on Miettinen and Lasota’s [8] Armed Forces Institute of Pathology (AFIP) stratification [9]. Although these systems are useful in predicting GIST behavior, it is based on the assumptions of a wide range of experts on GISTs.

KIT mutations were reported to associate with tumor metastasis and poor clinical outcome in GISTs [10]. On the contrary, PDGFRA mutations were typically characterized by clinically benign tumors [11,12]. Furthermore, the mutational status of KIT and PDGFRA is also a significant predictive factor for response to imatinib [13]. KIT and PDGFRA mutations appear to be related to outcome, but have not yet been integrated into the risk classification schemes. Previous studies were unable to distinguish the potential value of primary gene alterations in the risk of malignant biological behavior among gene subgroups or in controls to potentially confirm the variables examined [3,14-30]. This was possibly due to small sample sizes or confounding variables.

Therefore, we initiated an international collaborative effort which resulted in a meta-analysis of data on individual patients in prospective cohort studies to evaluate the prognostic value of KIT/PDGFRA mutations in GIST. To supply more powerful evidence, not only the tumor size, mitotic count, and tumor site, which have been used as parameters in NCCN criteria, but also clinical follow-up results such as recurrence, metastasis, and overall survival were tabulated and analyzed in our study.

## Methods

### Publication search

**Figure 1:**
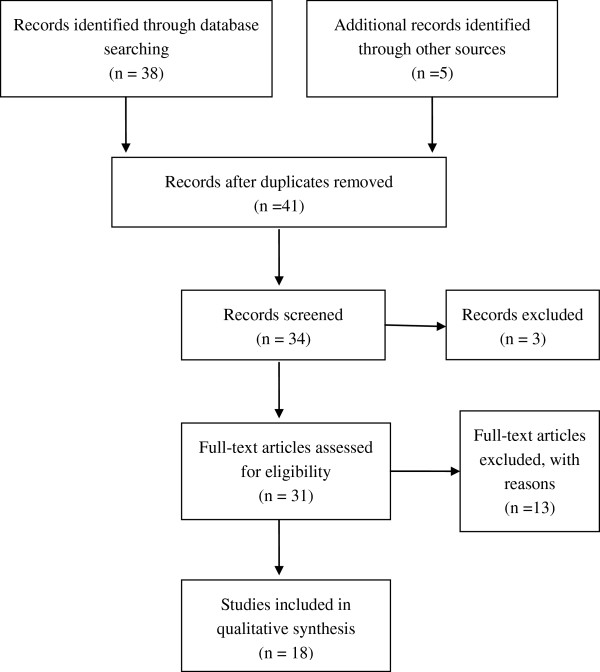
Flow chart of literature selection.

Two electronic databases (PubMed and Embase) were searched (last search was updated on 1 May 2012), using the search terms: ‘gastrointestinal stromal tumor’ and ‘KIT/PDGFRA mutation’. All eligible studies were retrieved, and their bibliographies were checked for other relevant publications. Review articles and bibliographies of other relevant studies identified were hand-searched to identify additional eligible studies. Only published studies with full-text articles were included. When the same patient population was included in several publications, only the most recent or complete study was used in this meta-analysis (Figure 1).

### Inclusion criteria

The inclusion criteria were as follows: 1) KIT/PDGFRA mutations and prognosis; 2) KIT/PDGFRA mutations in primary tumor before the treatment of imatinib; and 3) sufficient published data (more than 20 cases) to estimate an odds ratio (OR) with 95% confidence interval (CI).

### Data extraction

Information was carefully extracted from all eligible studies by two of the authors (LZ and PC), according to the inclusion criteria. The following data were collected from each study: study design (cohort, case-control, or cross-sectional), study population, sample size, total number of patients with positive KIT mutations and negative KIT mutations, and number of patients divided by age, gender, KIT expression, cell type, primary site, tumor size, mitotic count, recurrence, metastasis, and 3-year overall survival in those with and without KIT mutations, respectively.

### Statistical analysis

The ORs with 95% CI were used to assess the predictive value of KIT mutations on the malignant risk of GISTs, according to the method of Woolf. Heterogeneity assumption was confirmed by the χ^2^-based Q-test. A *P* value greater than 0.10 for the Q-test indicated a lack of heterogeneity among the studies, therefore the OR estimate for each study was calculated by the fixed effects model (the Mantel-Haenszel method). Otherwise, the random effects model (the DerSimonian and Laird method) was used. The significance of the pooled OR was determined by the Z-test and *P* >0.05 was considered statistically significant. Sensitivity analyses were carried out to determine if modification of the inclusion criteria for this meta-analysis affected the final results. An estimate of potential publication bias was carried out using the funnel plot, in which the OR for each study was plotted against its log (OR). An asymmetric plot suggested possible publication bias. Funnel plot asymmetry was assessed using Egger’s linear regression test, a linear regression approach to measure funnel plot asymmetry on the natural logarithm scale of the OR. The significance of the intercept was determined by the *t*-test, as suggested by Egger (*P* <0.05 was considered representative of statistically significant publication bias). All statistical tests were performed with Review Manager, version 4.2 (The Cochrane Collaboration, Oxford, UK) and STATA, version 9.2 (Stata Corporation, College Station, TX, USA).

## Results

### Study characteristics

A total of 43 publications met the inclusion criteria [3,10,14-54]. A series of studies with single factor analysis of KIT gene mutations were excluded due to lack of controlled gene subgroups [10,31-36]. Studies by Wardelmann *et al.* and Koyama *et al.* were also excluded because they screened metastatic GIST patients specializing in secondary KIT mutations under treatment with imatinib [37,38]. In addition, the study by Kikuchi *et al.* was excluded because it focused on heterozygosity as a useful post-recurrence prognosis in screened patients with liver metastasis [39]. The studies by Zheng *et al.* were also excluded because the included articles contained the same patient population [40,41]. Other studies were excluded due to insufficient information to calculate OR [42-54]. Hence, a total of 18 studies including 1,487 patients were used in the pooled analyses. Table 1 lists the studies identified and their main characteristics. Of the 18 groups, the sample size ranged from 25 to 177.

**Table 1:** Main characteristics of all studies included in the meta-analysis

**Study**	**District**	**Study period**	**Study size**	**Age (mean, years)**	**Gender (male/female)**	**Subgroups**	**Prognostic system**	**Follow-up time (mean, years)**
Daniels [3]	Germany	2011	87	64.9	45/42	KIT/PDGFRA/BRAF/WT	NCCN risk	NA
Taniguchi [14]	Japan	1999	124	60	NA	KIT positive/negative	BM	4.1
Sakurai [15]	Japan	1999	48	59.4	21/27	KIT positive/negative	BM	3.7
Yamamoto [16]	Japan	2004	27	59	15/24	KIT/PDGFRA/WT	M-MIB index	3.6
Lin [17]	Taiwan	2006	25	63.2	13/12	KIT positive/negative	NA	NA
Kim [18]	Korea	2004	86	59.5	47/39	KIT positive/negative	NIH risk	NA
Liu [19]	China	2005	82	53	56/26	KIT positive/negative	BM	4.1
Tzen [20]	Taiwan	2007	134	NA	74/60	KIT/PDGFRA/WT	NA	3.9
Cho [21]	Japan	2006	56	61	35/21	KIT/PDGFRA/WT	BM	4.7
Keun [22]	Korea	2008	68	56	31/37	KIT/PDGFRA/WT	NIH risk	5.0
Andersson [23]	Sweden	2006	177	NA	NA	KIT/PDGFRA/WT	MTR-KI67 index	6.2
Haller [24]	Germany	2005	38	64	22/16	KIT/PDGFRA/WT	NIH risk	2.7
Steigen [25]	Norway	2007	89	64.8	50/39	KIT/PDGFRA/WT	BM	NA
Zheng [26]	China	2011	25	58	15/10	KIT/PDGFRA/WT	MC-KI67 index	3.2
Wardelmann [27]	Germany	2003	55	62	29/26	KIT/PDGFRA/WT	BM	NA
Martín [28]	Spain	2005	162	63	82/80	KIT positive/negative	NIH risk	3.5
Penzel [29]	Germany	2005	79	60.9	41/38	KIT/PDGFRA/WT	NIH risk	NA
Agaram [30]	America	2006	125	NA	NA	KIT/PDGFRA/WT	NA	NA

BM, benign to malignant; NA, not available; NCCN, National Comprehensive Cancer Network; NIH, National Institutes of Health; MC-KI67 index, mitotic count and Ki-67 index; M-MIB index, mitotic rate and MIB-1 index; MTR-KI67 index, maximum tumor size and Ki67 index; WT, wild type.

### General and pathological outcomes

**Figure 2:**
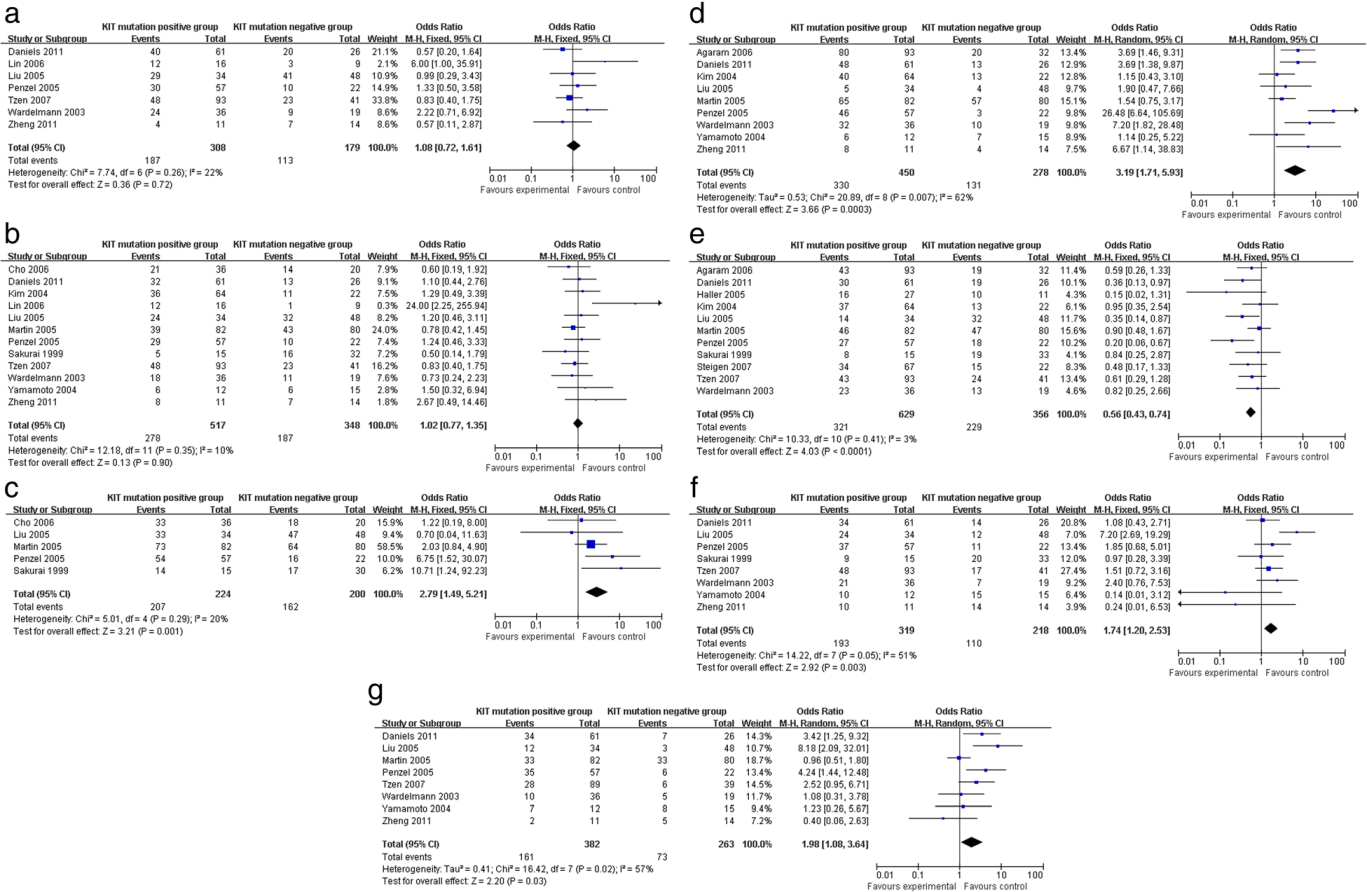
**General and pathological outcomes of KIT mutation-positive subgroup versus KIT mutation-negative subgroup. (a)** Age; **(b)** gender; **(c)** KIT expression; **(d)** cell type; **(e)** primary tumor site; **(f)** tumor size; and **(g)** mitotic count.

The meta-analysis of both age distribution and gender in the KIT mutation-positive versus -negative subgroups did not attain statistical significance (1.08 (95% CI, 0.72 to 1.61; *P* = 0.72) and 1.02 (95% CI, 0.77 to 1.35; *P* = 0.90), respectively) (Figure 2a,b). The overall OR for KIT expression in the KIT mutation-positive versus -negative subgroups was 2.79 (95% CI, 1.49 to 5.21; *P* = 0.001) (Figure 2c). The overall OR for spindle cells in the KIT mutation-positive versus -negative subgroups revealed a significantly elevated risk in the KIT mutation-positive subgroup, but for the stomach as a primary site, this was seen in the KIT mutation-negative subgroup (3.19 (95% CI, 1.71 to 5.93; *P* = 0.0003) and 0.56 (95% CI, 0.43 to 0.74; *P* <0.0001), respectively) (Figure 2d,e). However, an increased risk for larger tumor size (>5 cm) and higher mitotic activity (>5) was observed in the KIT mutation-positive subgroup (1.74 (95% CI, 1.20 to 2.53; *P* = 0.003) and 2.00 (95% CI, 1.08 to 3.68; *P* = 0.03), respectively) (Figure 2f,g).

### Clinical outcomes

**Figure 3:**
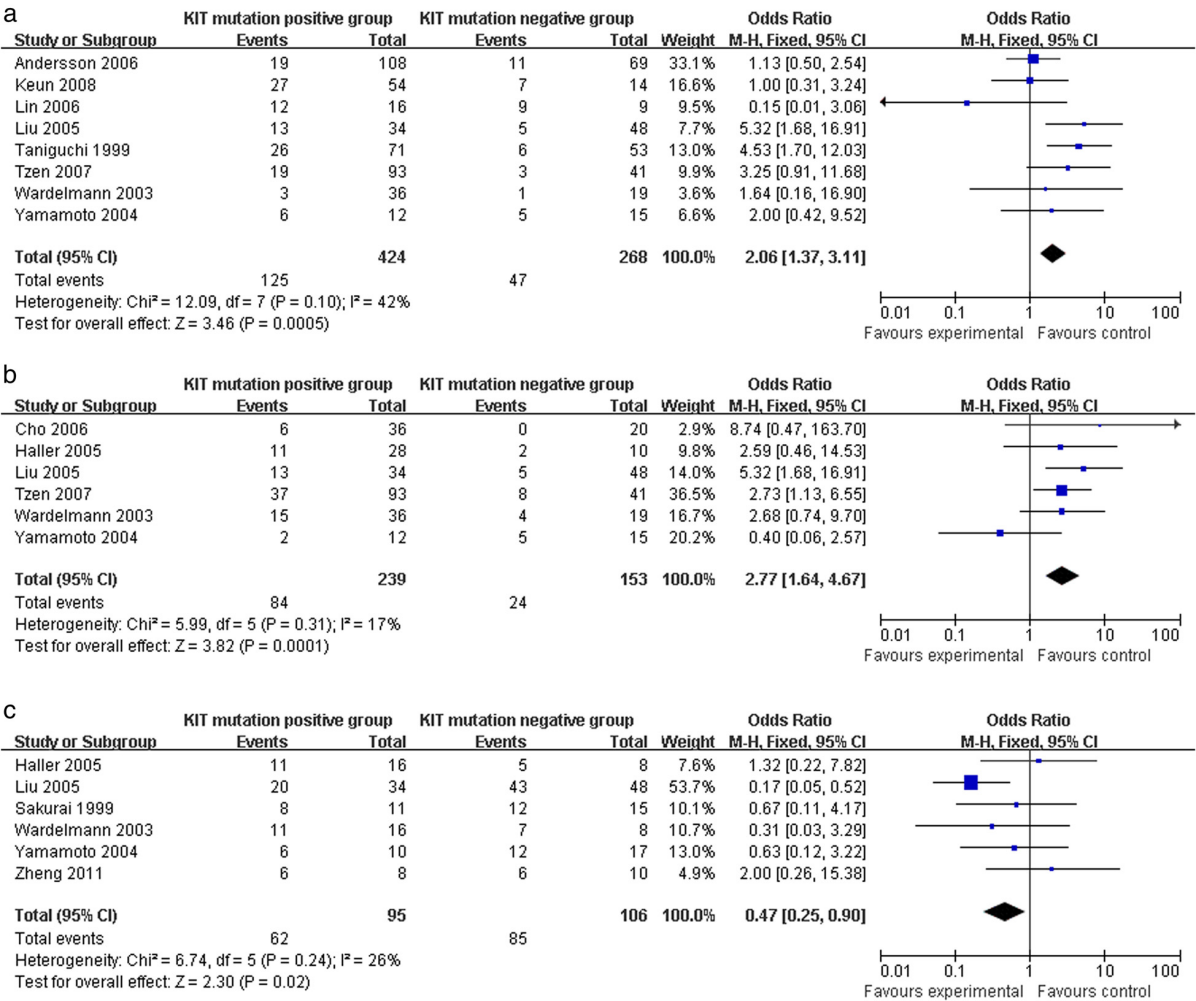
**Clinical outcomes of KIT mutation-positive subgroup versus KIT mutation-negative subgroup. (a)** Recurrence; **(b)** metastasis; and **(c)** 3-year overall survival.

The KIT mutation-positive subgroup showed a significantly higher rate of recurrence and metastasis compared to the KIT mutation-negative subgroup (2.06 (95% CI, 1.37 to 3.11; *P* = 0.0005) and 2.77 (95% CI, 1.64 to 4.67; *P* = 0.0001), respectively) (Figure 3a,b). Moreover, KIT mutations demonstrated a worse prognosis in high risk or malignant GISTs, which was supported by the 3-year overall survival analysis (OR 0.47 (95% CI, 0.25 to 0.90; *P* = 0.02)) (Figure 3c).

### Publication bias

Begg’s funnel plot was performed to assess publication bias. The heterogeneity tests for comparing the 18 combined studies showed heterogeneity in some analyses such as cell type and mitotic count; however, significant heterogeneity among the studies was not found (Table 2). No single study influenced the pooled OR qualitatively as indicated by the sensitivity analyses (data not shown).

**Table 2:** Outcomes of the meta-analysis

**Parameter**	**Included studies**	**Sample size**		**Heterogeneity**	**OR**	**95% CI of overall effect**	***P *** **value**
		**KIT mutation-positive**	**KIT mutation-negative**				
Age (≥40 years)	7	308	179	*P* = 0.26, I^2^ = 22.4%	1.08	0.72 to 1.61	*P* = 0.72
Gender (male)	12	517	348	*P* = 0.35, I^2^ = 9.7%	1.02	0.77 to 1.35	*P* = 0.90
KIT expression	5	224	200	*P* = 0.29, I^2^ = 20.2%	2.79	1.49 to 5.21	*P* = 0.001
Cell type (spindle cell)	9	450	278	*P* = 0.007, I^2^ = 61.7%	3.19	1.71 to 5.93	*P* = 0.0003
Primary tumor site (stomach)	11	629	356	*P* = 0.41, I^2^ = 3.2%	0.56	0.43 to 0.74	*P* <0.0001
Tumor size (>5 cm)	8	319	218	*P* = 0.05, I^2^ = 50.8%	1.74	1.20 to 2.53	*P* = 0.003
Mitotic count (>5)	8	382	263	*P* = 0.02, I^2^ = 57.8%	2.00	1.08 to 3.68	*P* = 0.03
Recurrence	8	424	268	*P* = 0.10, I^2^ = 42.1%	2.06	1.37 to 3.11	*P* = 0.0005
Metastasis	6	239	153	*P* = 0.31, I^2^ = 16.5%	2.77	1.64 to 4.67	*P* = 0.0001
3-year overall survival	6	95	106	*P* = 0.24, I^2^ = 25.8%	0.47	0.25 to 0.90	*P* = 0.02

## Discussion and conclusion

NIH and NCCN systems were established to predict GIST behavior using risk assessment (very low risk, low risk, intermediate risk, and high risk). However, it is still not clear whether internal molecular events correlate with malignant risk in GISTs. Molecular findings are critical in understanding the pathogenesis of GISTs. In 1998, Hirota *et al.* made the landmark discovery that the majority of GISTs harbor an activating mutation in the KIT oncogene [2]. Just 5 years later, Heinrich *et al.* identified oncogenic mutations in PDGFRA in a small subset of GISTs lacking KIT mutations, which meant that the mutations in PDGFRA and KIT were mutually exclusive [4]. Since then, subsequent evidence has shown that these mutations are pathogenetic for GIST initiation. However, between 10% and 15% of GISTs do not have KIT or PDGFRA mutations (known as wild type GISTs) and are a heterogeneous group, and in a recent study mutations in BRAF were found [3].

To date, many studies have focused on stratifying GISTs into prognostic categories based on mutational types, besides tumor size, mitotic count, and tumor site. But these studies showed controversial results and raised high concern. For example, Taniguchi *et al.* showed that KIT mutation was an independent prognostic factor for overall and cause-specific survival in patients with GISTs, whereas Sakurai *et al.* and Yamamoto *et al.* failed to observe such an association [14-16]. Lin *et al.* suggested that KIT mutation, compared with PDGFRA mutation and wild type, was often found in male patients and was more frequently found in those with large GISTs; however, they did not determine the predictive value of KIT and PDGFRA mutations [17]. In contrast, Kim *et al.* suggested that KIT mutation, along with high mitotic count and larger tumor size, had a strong prognostic value [18]. Prospective data have been criticized as being less convincing due to small sample size and the lack of statistical power to integrate sporadic individual studies. With a goal to explore the prognostic value of KIT/PDGFRA mutations, we performed this meta-analysis to derive an overall pooled estimation of published studies. Since KIT mutations were proportionally more frequent than PDGFRA mutations and wild type, we divided all GISTs into KIT mutation-positive and -negative subtypes (PDGFRA mutations and wild type).

In analysis of mutation subtypes and biological behavior of GISTs, the results were as follows: 1) KIT mutations correlated with higher KIT expression level than PDGFRA mutations and wild type; 2) KIT mutations had a preference for spindle cell in histology; 3) PDGFRA mutations and wild type more frequently occurred in the stomach; and 4) KIT mutations showed a marked increased risk in both larger tumor size (>5 cm) and higher mitotic count (>5), which revealed that KIT mutations significantly correlated with NCCN high risk or NIH high risk.

Given these histopathological findings, we also examined the relationship between mutation subtypes and clinical follow-up outcomes, which revealed that KIT mutations correlated with higher malignant risks than PDGFRA mutations and wild type, mainly because of: 1) more frequent recurrences; 2) higher metastasis; and 3) a worse survival rate.

It is very interesting that KIT mutations in high risk or malignant GISTs represent a worse factor of prognosis than PDGFRA mutations and wild type based on our findings, but patients with KIT mutations benefit a lot from the targeted therapy of imatinib. On reviewing the latest studies, our previous results supported that patients with KIT mutations have improved response to imatinib treatment when compared with those with wild type. However, the long-term efficacy is not significant [13]. Moreover, patients with KIT mutations who initially benefit from imatinib treatment eventually develop drug resistance. Recent studies reported that those patients with secondary imatinib resistance are through polyclonal acquisition of second-site mutations in the kinase domain. Regardless of this, it has been proved that constitutive KIT/PDGFR activation promotes proliferation and inhibits apoptosis of neoplastic cells through the CCRP signaling pathway [55]. An alteration in CCRP is often implicated in the pathogenesis and tumor progression of various types of tumors. Therefore, secondary mutation and CCRB signaling pathway might be the possible mechanism to explain the discrepancy of KIT mutations in prognosis and target therapy.

## Abbreviations

AFIP: Armed Forces Institute of Pathology; CI: Confidence interval; GIST: Gastrointestinal stromal tumor; NCCN: National Comprehensive Cancer Network; NIH: National Institutes of Health; OR: Odds ratio.

## Competing interests

The authors declare that there is no conflict of interest with regard to the following: employment, consultancies, stock ownership, honoraria, paid expert testimony, patent applications/registrations, and grants or other funding.

## Authors’ contributions

LZ and PC contributed equally to this manuscript. Both authors read and approved the final manuscript.
